# Fractals Analysis of Cardiac Arrhythmias

**DOI:** 10.1100/tsw.2005.81

**Published:** 2005-09-06

**Authors:** Mohammad Saeed

**Affiliations:** Department of Biological and Biomedical Sciences, The Aga Khan University, Karachi, Pakistan

**Keywords:** fractals, chaos, heart, rhythms, aging, cardiovascular disease, noninvasive, arrhythmia

## Abstract

Heart rhythms are generated by complex self-regulating systems governed by the laws of chaos. Consequently, heart rhythms have fractal organization, characterized by self-similar dynamics with long-range order operating over multiple time scales. This allows for the self-organization and adaptability of heart rhythms under stress. Breakdown of this fractal organization into excessive order or uncorrelated randomness leads to a less-adaptable system, characteristic of aging and disease. With the tools of nonlinear dynamics, this fractal breakdown can be quantified with potential applications to diagnostic and prognostic clinical assessment. In this paper, I review the methodologies for fractal analysis of cardiac rhythms and the current literature on their applications in the clinical context. A brief overview of the basic mathematics of fractals is also included. Furthermore, I illustrate the usefulness of these powerful tools to clinical medicine by describing a novel noninvasive technique to monitor drug therapy in atrial fibrillation.

## INTRODUCTION

Change is the only constant. This is especially true for biological systems and processes, which provide an astonishing source of complexity and flux. Yet clinical medicine has been dominated by the concept of homeostasis, which aims to normalize measured values and reduce variability[1]. Heart rhythms are a typical example. Close examination of the "regular" sinus rhythm reveals extraordinary variability even in resting subjects. The temporal fluctuations in sinus rhythm have complex dynamics involving multiple scales of time from milliseconds to hours. Contrary to contemporary belief, these fluctuations hold vast information content; however, traditional measures of data analysis, such as means and variances, ignore these data as uninformative "noise".

Irregular processes that appear to be random, but are in fact governed by an underlying hidden set of rules, are said to be in a state of chaos. Chaotic systems are characterized by complex dynamics, determinism (i.e., nonprobabilistic, in the sense that the value of the system at the previous point determines the value of the current signal according to some set of rules), and sensitivity to initial conditions. If the precise sets of rules determining the irregularity in the rhythms are well understood, it can be predicted, eliminated, or controlled. Wherever chaotic processes are at play, they lead to the formation of a fractal structure. Fractals are geometric objects that can be divided into parts, each of which is similar to the original whole. They are, therefore, self-similar objects independent of scale and possessing infinite detail. Application of the tools of chaos theory (nonlinear dynamics) to heart rhythms has revealed their fractal nature. The "noise" is beginning to be understood. More importantly, it has been shown that the collapse of this fractal organization into excessive order or uncorrelated randomness leads to a less-adaptable system and is a characteristic of aging and disease. With the tools of nonlinear dynamics, this fractal breakdown can be quantified with potential applications to diagnostic and prognostic assessment.

In this review, I describe the methodologies for analysis of cardiac rhythms and their background. I also hope to provide the reader with a simple, yet comprehensive, appraisal of the basic mathematics of fractals to encourage understanding of their simplistic principles and wide-ranging applications. The current literature on the applications of fractal analysis to atrial fibrillation as an example of heart rhythms is reviewed. Furthermore, I illustrate the usefulness of these tools to the clinician by describing a novel noninvasive technique to monitor drug therapy in atrial fibrillation. With all this, I intend to present before the audience the extraordinary power of fractal analysis and encourage its rapid dissemination into clinical practice.

## FRACTALS: THE CONCEPT

Benoit Mandelbrot, the father of fractals, coined the term in 1975 from the Latin word *fractus*, meaning broken or irregular[2]. Working at IBM in New York, he published a paper in 1963 that is now considered a classic on the fluctuations of cotton prices[3]. Previously, economists believed that price changes followed Gaussian distributions, making the possibility of large variations rare. Mandelbrot observed that the pattern of cotton prices was similar irrespective of the time period of study, i.e., the pattern of peaks and troughs looked the same over a week as it did over a year. He showed that this scale-free, "self-similar" behavior followed a different distribution, known as the power law. The law predicts that large variations are, in fact, common[4].

Fractals can be generated by a repeating pattern coded mathematically by a recursive or an iterative process. They are born of complex numbers, which are sums of real and imaginary numbers. Fractals may be classified according to their self-similarity, which may be Exact, Quasi-self-similar, or statistically self-similar. *Exact* fractals are generated by iterative functions (e.g., Koch's snowflake). *Quasi*-self-similar fractals, defined by recurrence relations, display a relatively loose form of self-similarity where the fractal appears approximately (but not exactly) identical at different scales (e.g., Mandelbrot set). Quasi-self-similar fractals contain small copies of the entire fractal in distorted and degenerate forms. *Statistically* self-similar fractals have numerical or statistical measures that are preserved across scales of time or space (e.g., heart rhythms, arterial networks).

If a Euclidean object such as a square of length $L$ is cut into $N$ number of smaller squares with length $l$, then the relationship between these variables is defined by the equation $\mathrm{N} = \mathrm{r}^{\mathrm{D}}$, where r is the ratio of $L$ to $l$ and $D$ is the *fractal dimension* of the object[5]. It can be any non-negative real number including a fraction, unlike the topological dimension used to describe Euclidian objects, which is always an integer. Fractal dimension is a numerical measure that is preserved across scales and, thus, can be used to define fractal objects. However, when fractal objects such as the Koch's snowflake or coastlines of countries are measured, their apparent length is dependent on the length of the measuring stick $l$. The smaller the stick, the more accurate the measurement. Mandelbrot defined this relationship by the equation[6]:

$$\ln \mathrm{L}(l) = 1\text{-}\mathrm{D} \ln l + \mathrm{c}$$

where $\mathrm{L}(l)$ is the estimated overall contour length of the structure using a stick of length $l$ and c is the y-intercept of the equation on a log-log graph. The derivation of this equation has been elaborated elsewhere[7]. The graph results in a straight line with a negative slope equal to 1-D. Using this method,

Mandelbrot showed that coastlines, like Koch’s snowflake, are fractal structures possessing statistical self-similarity[6].

## FRACTALS ANALYSIS IN BIOLOGY

Biologic systems exhibit extraordinary complexity in both space and time dimensions. Thus, the nonlinearity and nonstationarity of processes in living organisms defy conventional mathematical and biostatistical methodologies of study. By providing a geometric framework for the description of apparently irregular patterns, fractal analysis is able to characterize natural structures[8]. In the human body, several structures are fractal in nature, such as the arterial and venous trees, neural networks, tracheobronchial architectures, and the His-Purkinje system[9]. These self-similar cardiopulmonary and neural structures serve at least one fundamental physiologic function: rapid and efficient transport over complex, spatially distributed networks. The fractal concept can be applied not only to irregular geometric forms that lack a characteristic (single) scale of length, but also to certain complex processes that lack a single scale of time. Fractal processes generate irregular fluctuations across multiple time scales, analogous to the arterial branching networks that have multiple scales of length[10]. A qualitative appreciation for the self-similar nature of fractal processes can be obtained by plotting their fluctuations at different temporal resolutions (Fig. 1).

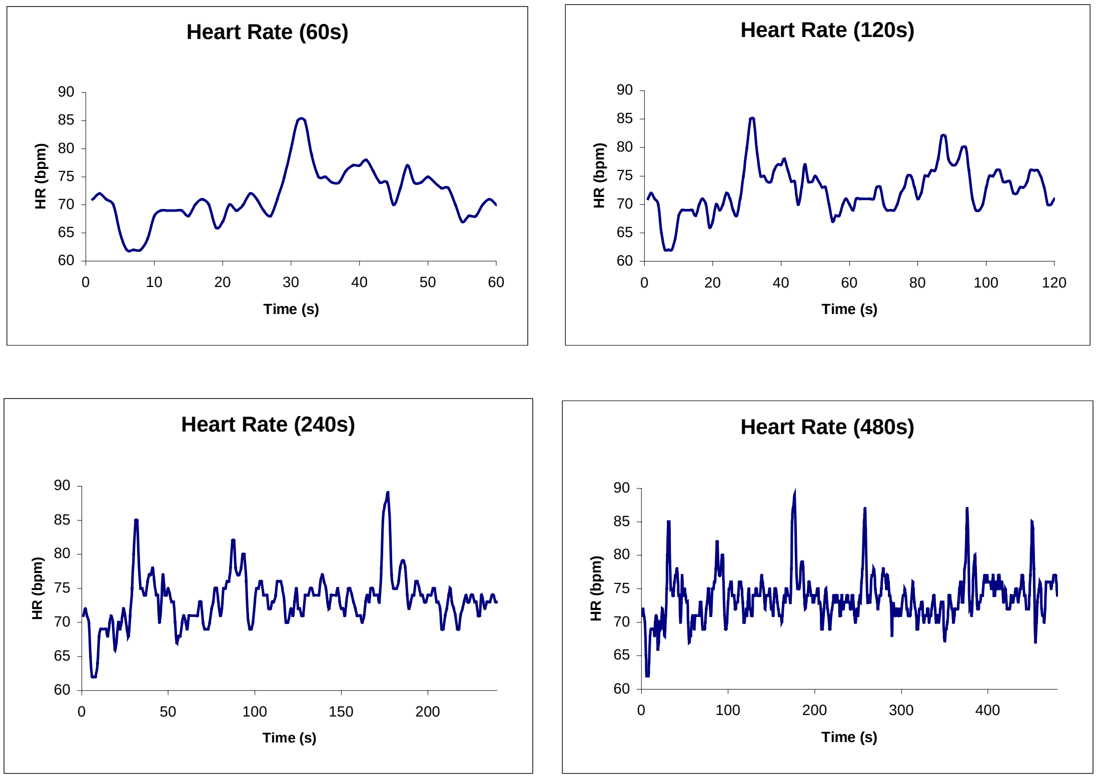

**FIGURE 1.** Heart rate of a healthy individual over time scales in increments of a factor of 2. Notice the similarity in the tracings to each other and the basic 60s tracing highlighting the self-similar nature and temporal correlation of heart rate dynamics. The data were digitally obtained using the Portapres system (48) at a frequency of 100 Hz. For ease of comparison, the y-axis is kept constant for all tracings (60–90 bpm).

This self-similarity can be quantified by measurements classified into two general classes: “moment” statistics and dynamic statistics[11,12,13]. The first class of measures, time-domain or moment statistics

(e.g., mean, standard deviation [SD] and coefficient of variation), is based on linear analysis. For heart rhythm variability analysis, SDNN is another time-domain measure, which quantifies the SD of all normal-to-normal RR intervals[14]. All of these measures are relatively straightforward to obtain (although careful attention to erroneous or missing data is necessary because they tend to be highly sensitive to isolated outliers). Such measures do not depend on the order of the observations, however, and may therefore obscure significant information about heart rhythm dynamics. For example, two heart rate time series may have nearly identical mean rates and SDs, but very different dynamics[15,16,17].

Dynamic statistics, a class that includes both frequency-domain measures, such as power spectral density estimates derived from Fourier analyses and new measures derived from nonlinear dynamics, do preserve information about the order of observations. The Fast Fourier transform (FFT) provides a useful representation of the component frequencies of heart rate time series: ultra low frequency (ULF; <0.0033 Hz), very low (VLF; 0.0033–0.04 Hz), low (LF; 0.04–0.15 Hz), and high frequency (HF; 0.15–0.40 Hz). The gradient (exponent $\beta$) of the log (power) on log (frequency) plot in the frequency range of $10^{-4}$–$10^{-2}$ provides a measure of the power law scaling (1/f) of R-R intervals[18,19]. Nonstationarity in typical heart rate time series severely limits the range of frequencies that can be studied by conventional frequency-domain analytic methods. Furthermore, frequency-domain analysis, while retaining information relating to ordering of observations, is still based on linear models and may conceal the details of interactions between mechanisms. Nonlinear and fractal dynamic analysis offers the prospect of revealing these details by providing direct measures of complexity[20,21] and long-range correlations[22,23]. Following is a brief description of some of the methods in use today.

## Relative Dispersion Analysis (RDA)

This method employs Mandelbrot's equation ($\ln L[l] = 1\text{-}D \ln l + c$) to quantitate the self-similarity in a time series. The stick length $l$ is represented by a time period, referred to as a window. By varying the window size, as in Fig. 2, and plotting the variation in the signal against the window size on a log-log scale quantifies the fractal dimension (D). Details of the method have been described previously[7]. Briefly, if there are 1000 data points for a heart rate recording, the fractal dimension can be estimated by calculating the ratio of SD to the mean for 1000 points (window size). In this case, the entire dataset is a single window. Decreasing the data points included in each window can increase the number of windows. Conventionally, the window size (n) is increased in a power-law fashion with base 2, i.e., $2^n$. Thus, for a dataset of 1000 points, there can be window sizes of 2, 4, 8, 16, 32, 64, 256, and 512 (up to $2^8$). This means that for a window size of 256, the mean of sets of 256 points (3 complete sets in 1000 point dataset) will be calculated. This set of means (3 in number) is used to define an overall mean and SD of this series. The log of the ratio of SD to the mean in percent (SD*100/mean), called the coefficient of variation, of this series is plotted against the log of the window size (n = 7). This process is repeated for all window sizes possible for a dataset. Linear regression of all points on the graph results in the line-of-best-fit. The gradient (1-D) of this line on the log-log graph substracted from 1 is the fractal dimension of the time series (Fig. 2A).

## Detrended Fluctuation Analysis (DFA)

Another method for quantitative analysis of self-similarity in a time series is Detrended Fluctuation Analysis (DFA), first proposed to estimate long-range correlations in nucleotide sequences in DNA[24]. The original time series is integrated and then divided into boxes of equal length. For each box, a least squares line (representing the *trend* in that box) is fit to the data. The characteristic size of the fluctuations in each box, denoted by $F(n)$, is calculated as the root mean square deviation from the trend.

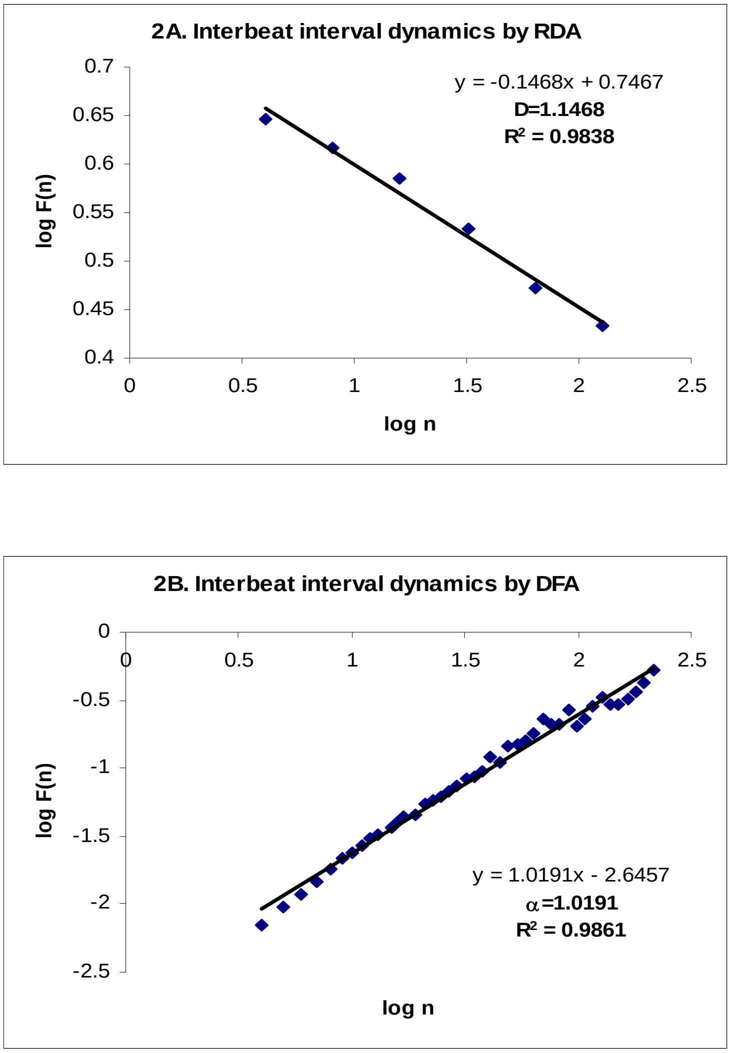

**FIGURE 2.** The interbeat interval (R-R) time series of the heart rate recording shown in Fig. 2 is subjected to fractal analysis using RDA and DFA. Both methods demonstrate fractal structure of the heart rhythm with fractal dimensions (D) and long-term scaling exponent close to 1.0 and strong regression coefficients ($R^2$ > 0.98).

This computation is repeated over all time scales (box sizes). Typically, *F(n)* will increase with box size *n*. A linear relationship on a log-log graph indicates the presence of scaling (self-similarity), such that fluctuations in small boxes are related to the fluctuations in larger boxes in a power-law fashion. The slope of the line relating log *F(n)* to log *n* determines the fractal scaling exponent, α (Fig. 2B). This exponent provides a measure of the "roughness" of the original time series: the larger the value of α, the smoother the time series. In this context, 1/*f*-like noise (α = 1) can be interpreted as a "compromise" between the complete unpredictability of white noise (α = 0.5) and the much smoother "landscape" of Brownian noise (α = 1.5)[9,25]. It has been observed that the DFA plot may not be strictly linear, but rather consist of two distinct linear regions of different slopes separated by a breakpoint. Thus, some studies characterize α as short-term scaling exponent (4–11 beats) and long-term exponent (>11 beats)[26]. Correlations between DFA and Fourier analysis have also been described[27]. Due to detrending on all time scales, DFA is robust against artifacts of nonstationarities. Thus, it does not require data clean-up to remove ectopic beats or measurement artifacts that introduce a subjective bias in the analysis. It can therefore be automated and applied to “free running” ambulatory conditions[17].

### Other Fractal Measures

Several other fractal measures have been developed, each with its own advantages and limitations. Approximate Entropy (APEN) estimates the log-likelihood that the next R-R intervals after each set of patterns will differ (i.e., the similarity of the patterns is coincidental and lacks predictive value)[28]. Large values of APEN indicate greater irregularity of the time series. Wavelet-based nonlinear analysis has been shown to elucidate phase interactions between the different frequency components of heart rhythms in addition to long-range correlations[29,30]. Chaotic test is a measure robust to random noise that can differentiate chaos from stochasticity[31]. A full description of these and other methods is beyond the scope of this review.

## RHYTHM ANALYSIS OF ARRHYTHMIAS

Ventricular fibrillation (VF) is the most common initial arrhythmia associated with sudden cardiac death, which accounts for one-half of all coronary heart disease-related deaths[32]. Yet there is little information on specific markers of arrhythmic death and in spite of large epidemiological surveys[33,34,35,36]. A large prospective study in the elderly (>65 years), general population of Finland revealed that the short-term scaling exponent of DFA was associated with mortality, especially of cardiac origin (relative risk = 2.5)[37]. In cases with sudden cardiac deaths (n = 29), the short-term fractal exponent was 0.92 ± 0.19 compared to 1.14 ± 0.17 in survivors ($p < 0.001$). Traditional methods of heart rate variability have failed to predict imminent VF[37,38,39], however, the short-term fractal exponent was greatly reduced prior to onset of VF (0.64 ± 0.19) in patients with healed MI tested for induction of ventricular arrhythmias using programmed electrical stimulation[38]. Power-law regression slope $\beta$ was also decreased in this group of patients, whereas the HF spectral component was increased. In patients with ventricular tachycardia (VT), the short-term fractal exponent was also reduced (0.85 ± 0.25)[40]. This reduction correlated with periods of abrupt change in sinus cycle lengths evident as sinus pauses or alternating changes in interbeat intervals (IBI) without a change in P-wave morphology[40]. These observations imply the loss of fractal stability of heart rhythm dynamics in ventricular arrhythmias that is most pronounced before the onset of VF. The reduction in fractal exponent indicates a predominance of random-like form of heart rhythms in VT, VF, and sudden cardiac death. These abnormal IBI dynamics may not be causative for the arrhythmias, but may rather reflect changes in regulatory systems preconditioning the heart to abnormal rhythmicity.

Atrial fibrillation (AF) is the most common sustained cardiac arrhythmia[41]. It has been observed that prior to the onset of AF, there is an increase in heart rate and ectopic beats[42,43]. Fractal analysis of 24-h ECG recordings of patients with paroxysmal AF showed that APEN and short-term scaling exponent were significantly reduced prior to the onset of AF[42,43]. Similar results were found for ectopic atrial tachycardias[44]. These findings indicate a reduction in complexity and of fractal correlations in IBI time series preceding onset of AF. These subtle alterations in heart rhythm dynamics could not be picked up by traditional time- and frequency-domain measures[42,43,44].

Predicting arrhythmias in the intensive care setting and monitoring drug therapy with antiarrhythmics remains a dilemma of today’s medicine. In managing AF for example, the main therapeutic strategies include rate control, termination of the arrhythmia (rhythm control), and the prevention of recurrences and thromboembolic events[45]. The optimal strategy for arrhythmia management, however, remains uncertain because the mechanism of action of most antiarrhythmics is not fully understood[41,46,47]. In the following section, I present an example of the clinical application of fractals in cardiology, describing a new and novel noninvasive technique to monitor AF, as well as follow-up drug response in AF patients.

The technique can be used for in-hospital patients to monitor their progress or for long-term clinical care on an outpatient basis. Applied on a large scale, this methodology could be a useful aid in the clinical management of AF and drug trials of antiarrhythmics.

## Noninvasive Arrhythmia Testing: A Case

The patient was a 34-year-old male, healthy and active, who presented with sudden onset palpitations of 1-day duration. He did not have any significant past medical history or family history of cardiovascular disease. He was not on any medication. Physical examination revealed an irregularly irregular pulse with tachycardia (130–180 bpm) and blood pressure of 115/70 mmHg. Cardiopulmonary examinations were otherwise within normal limits. An ECG revealed AF. Intravenous metoprolol (5 mg) was given which reduced the heart rate to 60–80 bpm. He was admitted for further investigations and management to the Special Care Cardiology Unit on oral atenolol 50 mg, once-daily dose. Cardiac enzymes turned out to be within normal limits and a serum TSH level of 1.22 IU/L (normal range 0.5–4.0 IU/L) was reported. Subsequently, an ECG was performed that reported normal cardiac structure and function (left atrial size, 36.3 mm) and a 24-h Holter recording showed an overall baseline sinus rhythm with occasional supraventricular premature complexes with an average heart rate of 68 bpm (range 43–105 bpm). Repeat ECGs on subsequent days of hospital admission showed disappearance of fibrillation and reversion to sinus rhythm. The patient was discharged with a diagnosis of idiopathic paroxysmal AF, on oral atenolol 50 mg once daily, and advised regular follow- up.

The patient, however, was noncompliant and stopped the medication on his own accord. He presented 2-weeks later to The Aga Khan University (Day 0) having noticed occasional palpitations for 2 days. His pulse was 75 bpm, regular, and blood pressure was 130/85 mmHg, but an ECG did not reveal AF. The patient was put on oral atenolol 50 mg once daily again and his heart rhythm was monitored regularly for 2 weeks, noninvasively on Portapres[48], for which an informed consent was taken.

The Portapres Model-2 (Institute of Applied Physics Biomedical Instrumentation, The Netherlands) is a finger-based, noninvasive, continuous, cardiovascular parameters' measurement and recording system[48]. Portapres recorded the IBI (R-R interval) digitally for 15 min at a frequency of 100 Hz and transferred the data to a microcomputer for fractal analysis. DFA was used to analyze this 15-min, cardiac, IBI time series to obtain the heart rhythm variability (HRV). As mentioned before, a linear relationship on a log-log plot indicated the presence of scaling, such that fluctuations on the small time scales were related to the fluctuations on the larger time scales in a power-law fashion. The slope of the line determined the fractal-scaling exponent, alpha ($\alpha$), which was used as a measure of HRV.

The patient had an alpha ($\alpha$) value of 0.66 at the start of the oral atenolol therapy (indicating a high degree of HRV) that increased to 0.97 after 12 days of once-daily dose of the drug. Thus, HRV showed progressive improvement towards normalization ($\alpha \approx 1.0$) with atenolol over time (Fig. 3). This study, therefore, also hints that atenolol may exhibit rhythm-controlling properties, which may be another mechanism of action of beta-blockers in AF.

Heart rate was the first to respond to therapy and decreased to 50–60 bpm after the initial dose of atenolol. The heart rate variance sharply dropped from 22.1 bpm$^2$ on Day 0 to 9.5 bpm$^2$ on Day 1 and then remained constant around this level until the end of the study. Beta-blockers are thought to mediate their effects on AF by controlling heart rate[47], however, the reduction in heart rate variance of the patient indicates an effect on heart rate variability as well. Both these effects, however, preceded the improvement in HRV ($\alpha$), demonstrating that rhythm control may be a delayed effect of beta-blockers. This report suggests that 15-min, nonambulatory, cardiac, IBI recordings followed by DFA may be a powerful technique to prognosticate AF and monitor drug therapy of patients in a noninvasive manner. Thus, temporal measurements of HRV could be useful tools in the clinical setting as well as trials of drugs in cardiovascular disease.

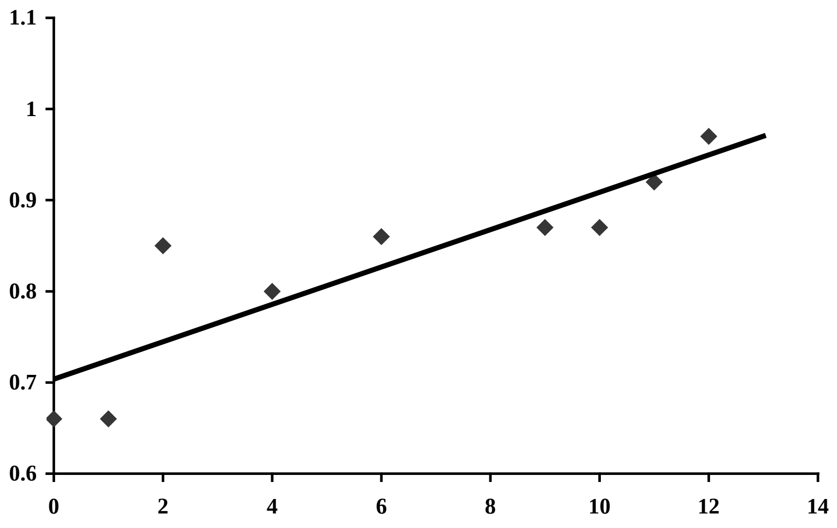

**FIGURE 3.** Plot of fractal scaling exponents ($\alpha$) against time (days), for a 34-year old patient with atrial fibrillation. Alpha values, inversely proportional to the degree of HRV, indicate a progressive increase ($\alpha$ = 0.02 t + 0.70; $R^2$ = 0.77; t = time in days) over a 12-day period of oral atenolol therapy. This provides evidence for the rhythm controlling mechanism of atenolol.

## CONCLUSIONS

Heart rhythms are generated by complex self-regulating systems that process inputs with a broad range of characteristics including determinism, long-range order, and sensitivity to initial conditions. Thus, they are inhomogeneous, nonstationary, and fluctuate in a complex manner. This complexity of heart rhythms is determined by chaotic processes, which originate from multiple oscillators coupled together in feedback loops[49]. The result of these processes is fractal organization of the emerging signals. Biomedical research has traditionally focused on analyzing individual components of a system and studying them in detail. Despite enormous success of this reductionist approach, it has significant limitations when characterizing chaotic systems, which amount to more than the sum of their parts. Fractal analysis provides appropriate measures for accurate estimation of the nonlinearity of chaotic systems. The application of these tools to cardiac physiology has unraveled the subtleties of heart rhythms in aging and disease. Furthermore, such studies have also revealed that the breakdown of fractal organization of heart rhythms into excessive order or uncorrelated randomness is a hallmark of disease and can be quantified using tools of nonlinear dynamics, with potential applications to clinical assessment and management.

## ACKNOWLEDGMENTS

The noninvasive study of atrial fibrillation was funded by the Department of Biological and Biomedical Sciences, The Aga Khan University, Karachi, Pakistan. I am extremely grateful to the patient, Mr. KA, for volunteering to participate in this study. I am also grateful to Professor H.R. Ahmad for introducing me to fractal analysis and for his support with the Portapres system. This work is lovingly dedicated to my parents, Mahmood and Naheed, for inspiring me and teaching me to discover knowledge.
